# InfectoCast: A Digital Ecosystem for Infectious Diseases Education and Science Communication in Brazil

**DOI:** 10.1093/ofid/ofag179

**Published:** 2026-03-27

**Authors:** Klinger Soares Faico-Filho, João Antonio Gonçalves Garreta Prats, William Dunke de Lima, Jordan Monteiro Pinheiro, Eusebio Lino dos Santos Junior, Carolina Larocca Santos

**Affiliations:** Departamento de Medicina, Disciplina de Clínica Médica e Medicina Laboratorial, Universidade Federal de São Paulo (UNIFESP), Escola Paulista de Medicina (EPM), São Paulo, SP, Brazil; InfectoCast, São Paulo, SP, Brazil; InfectoCast, São Paulo, SP, Brazil; InfectoCast, São Paulo, SP, Brazil; InfectoCast, São Paulo, SP, Brazil; InfectoCast, São Paulo, SP, Brazil; InfectoCast, São Paulo, SP, Brazil

## Abstract

**Background:**

Digital platforms have expanded access to continuing medical education (CME), with podcasts and social media increasingly used as asynchronous learning resources. However, most established digital medical education initiatives operate primarily in English, creating linguistic and contextual barriers for professionals in non-Anglophone settings. Infectious diseases (ID) require frequent updating, and access to structured ID-focused CME in middle-income countries such as Brazil remains uneven. InfectoCast is a Brazilian digital initiative that integrates podcasting, social media, and written resources to support infectious diseases education and science communication in Portuguese.

**Methods:**

We conducted a descriptive, retrospective quantitative analysis of platform-level analytics from the InfectoCast digital ecosystem between 18 September 2020 and 18 October 2025. Podcast metrics, including total plays, listening time, and geographic distribution, were extracted from Spotify for Podcasters. Social media metrics were obtained from native analytics dashboards, primarily from Instagram, with supplementary data from other platforms. Analyses focused on audience reach, growth trends, and the structure of the multi-platform dissemination strategy.

**Results:**

During the study period, the InfectoCast podcast released 168 episodes, accumulating 481 000 total plays and 53 962 hours of listening across 97 countries, with 96.9% of plays originating in Brazil. The Instagram account reached 110 000 followers and functioned as the primary platform for visual educational content, including infographics, educational carousels, short-form videos, and interactive Stories. When combined with YouTube, X (formerly Twitter), LinkedIn, the blog, and the newsletter, the ecosystem achieved an estimated monthly reach of approximately 3 million users. The integration of audio, visual, and written formats enabled coordinated dissemination of infectious diseases educational content across platforms.

**Conclusions:**

This descriptive case report documents the structure, reach, and growth of InfectoCast, a Portuguese-language digital ecosystem dedicated to infectious diseases education and science communication. The findings illustrate the feasibility of integrating podcasting with social media and other digital formats to deliver educational content in a non-Anglophone context. This ecosystem-based approach may inform the development of similar initiatives in other medical specialties and regions where access to locally contextualized digital education is limited.

Continuing medical education (CME) is essential for maintaining professional competence and improving the quality of healthcare delivery. However, traditional CME formats continue to face structural barriers, including limited time availability, high costs, and geographic constraints that disproportionately affect clinicians working outside major academic centers [1]. The digital transformation of medical education has introduced flexible, on-demand alternatives that partially mitigate these limitations [2]. Among these modalities, podcasts have emerged as a widely adopted format. A recent scoping review demonstrated that podcasts are a preferred asynchronous learning resource among trainees and are noninferior to traditional teaching methods for knowledge retention [3].

Infectious diseases (ID) represent a specialty in which continuous updating is particularly critical. Pathogen evolution, shifting antimicrobial resistance patterns, and frequent updates to clinical guidelines require ongoing access to reliable educational resources. In middle-income countries such as Brazil, access to structured and up-to-date ID education remains uneven and often fragmented, with high-quality resources concentrated in major academic institutions. This geographic and institutional centralization contributes to disparities in educational access for healthcare professionals practicing in underserved or remote regions.

In parallel, social media platforms have evolved beyond entertainment-oriented use and are increasingly incorporated into professional learning and scientific communication. Platforms such as Instagram, YouTube, X (formerly Twitter), and LinkedIn are commonly used by medical students, residents, and practicing clinicians to supplement formal education [4–6]. These platforms enable rapid dissemination of information, facilitate informal peer-to-peer learning, and provide opportunities for repeated exposure to educational content in concise formats.

The convergence of asynchronous audio learning, visual microlearning, and online professional networks has enabled the development of digital educational ecosystems that integrate multiple platforms and content formats. Such ecosystems provide multimodal learning environments in which audio, visual, and written materials are distributed across complementary channels, supporting reinforcement of key concepts and flexible engagement [7]. However, most established digital medical education initiatives operate primarily in English. For professionals in non-Anglophone settings, linguistic barriers, contextual mismatches, and limited incorporation of locally relevant epidemiological data reduce the applicability of these resources. In Brazil, these challenges are amplified by heterogeneous antimicrobial resistance patterns, endemic ID, and the structure of the national healthcare system, underscoring the need for context-specific educational approaches [8].

In response to these gaps, InfectoCast was created in 2017 as a collaborative educational initiative initiated by ID residents at the Universidade Federal de São Paulo. Initially developed as a podcast, the project progressively expanded into a multi-platform digital ecosystem dedicated to ID education and science communication in Portuguese. The ecosystem integrates long-form audio content with visual educational materials, a mobile application, a blog for extended discussions, a weekly newsletter, and an active presence across multiple social media platforms. Rather than focusing on translation of existing resources, the initiative emphasizes contextualization of content to the Brazilian epidemiological and clinical landscape.

The objective of this study is to provide a descriptive characterization of the InfectoCast digital ecosystem, with particular emphasis on its structure, platform integration, and audience reach. Through quantitative analysis of platform-level metrics, we aim to document how the combination of podcasting and social media-based dissemination can support scalable dissemination of ID educational content in a non-Anglophone context.

## METHODS

### Study Design

We conducted a descriptive, retrospective quantitative analysis of audience reach and engagement metrics across the InfectoCast digital education ecosystem. The study was designed as a descriptive case report of a digital educational initiative, focusing on platform-level analytics rather than on learner outcomes or clinical impact.

The observation period spanned during the most recent consolidated analysis period within the study timeframe, corresponding to the interval for which podcast and digital platform analytics were consistently available and comparable across time.

### Overview of the InfectoCast Digital Ecosystem

The InfectoCast ecosystem is structured as an integrated, multimodal environment for ID education and science communication. Although the podcast represents the core long-form educational format, the ecosystem is intentionally designed so that each digital platform fulfills a distinct and complementary role in content dissemination and reinforcement.

**Figure 1.**
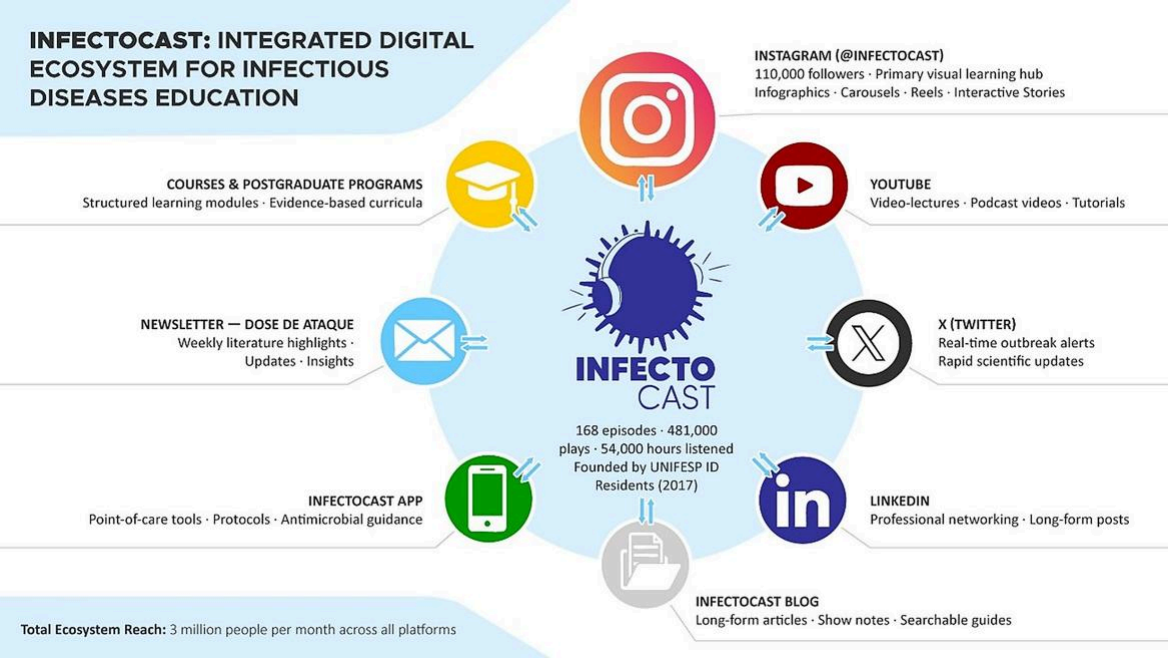
Structure of the InfectoCast digital ecosystem (Content Wheel). Schematic representation of the main components of the InfectoCast ecosystem and their complementary roles in content dissemination.

Content is specialized in ID and addresses topics relevant to clinical practice, public health, and antimicrobial use, with emphasis on conditions prevalent in the Brazilian healthcare context. Educational materials are delivered through multiple formats to support different learning preferences, including audio, visual, textual, and interactive modalities. The structure and interconnections of the InfectoCast digital ecosystem are illustrated in Figure 1.

The ecosystem comprises the following components:

1. *Weekly podcast* (*core educational format*)
  Audio episodes featuring expert interviews, case-based discussions, clinical reasoning, and evidence-based reviews. Podcast episodes serve as the foundational content from which other formats are derived.
2. *Instagram (@infectocast*)—*visual communication and community hub*
  The platform with the highest engagement, used for visual microlearning through infographics, educational carousels, short-form videos (Reels), and interactive Stories. Instagram also functions as a primary entry point for users into the broader ecosystem.
3. *YouTube*—*video-based content*
  Hosts video versions of podcast episodes, short educational videos, recorded lectures, and instructional series, expanding accessibility for users who prefer audiovisual formats.
4. X (*formerly Twitter*) and *LinkedIn*—*real-time updates and professional networking*
  X is used for rapid dissemination of scientific updates, news, and commentary related to ID. LinkedIn supports professional networking and longer-form posts aimed at clinicians and researchers.
5. *Blog/Website—long-form written repository*
  Provides searchable written content, including show notes, expanded clinical discussions, and educational articles optimized for online access.
6. *Support Tools—mobile application, newsletter, and educational courses*
  A mobile application offers point-of-care reference tools and clinical decision-support content. A weekly newsletter (“Dose de Ataque”) curates recent literature and highlights newly released content. Structured postgraduate courses provide formal continuing education opportunities within the ecosystem.

## DATA SOURCES AND COLLECTION

### Podcast Analytics

Podcast engagement metrics were extracted from Spotify for Podcasters, which accounted for the majority of listening activity during the study period. Collected metrics included total number of plays, listening hours, episode-level performance, device type, and geographic distribution of listeners. Data were exported in standardized monthly intervals to allow temporal analysis.

### Social Media and Digital Platform Analytics

Social media metrics were obtained using native analytics dashboards from each platform, including:

- *Instagram insights*: follower count, reach, impressions, and interactions (likes, comments, saves, and shares);
- *YouTube analytics*: total views, watch time, subscriber counts, and average view duration;
- *X (formerly Twitter) analytics*: impressions and engagement metrics;
- *LinkedIn analytics:* post impressions and follower growth.

For platforms with limited historical availability of analytics, consolidated internal records were used to supplement descriptive reporting.

### Newsletter Metrics

Newsletter performance data were obtained from the mailing platform used to distribute the weekly “Dose de Ataque” newsletter. Available metrics included number of delivered emails, open rates, and subscription changes for recent editions. Historical newsletter analytics were not consistently available for the entire study period and were therefore analyzed descriptively over the intervals for which data were accessible.

### Data Processing and Analysis

All data were collected, cleaned, and analyzed using Python version 3.11, with the Pandas library and standard visualization tools. Analyses were limited to descriptive statistics and included:

- Characterization of audience size and geographic distribution;
- Temporal trends in podcast plays and social media reach;
- Comparison of engagement metrics across platforms and content formats;
- Descriptive assessment of cross-platform dissemination patterns, such as temporal alignment between podcast episode releases and social media posts on related topics.

No causal inferences regarding educational effectiveness, changes in clinical behavior, or patient outcomes were performed.

### Ethical Considerations

This study relied exclusively on aggregated, platform-generated analytics data that were publicly available or provided through native dashboards. No individual-level data were collected, and no human subjects were involved. As such, ethics committee approval was not required.

## RESULTS

### Podcast Reach, Growth, and Audience Characteristics

**Figure 2.**
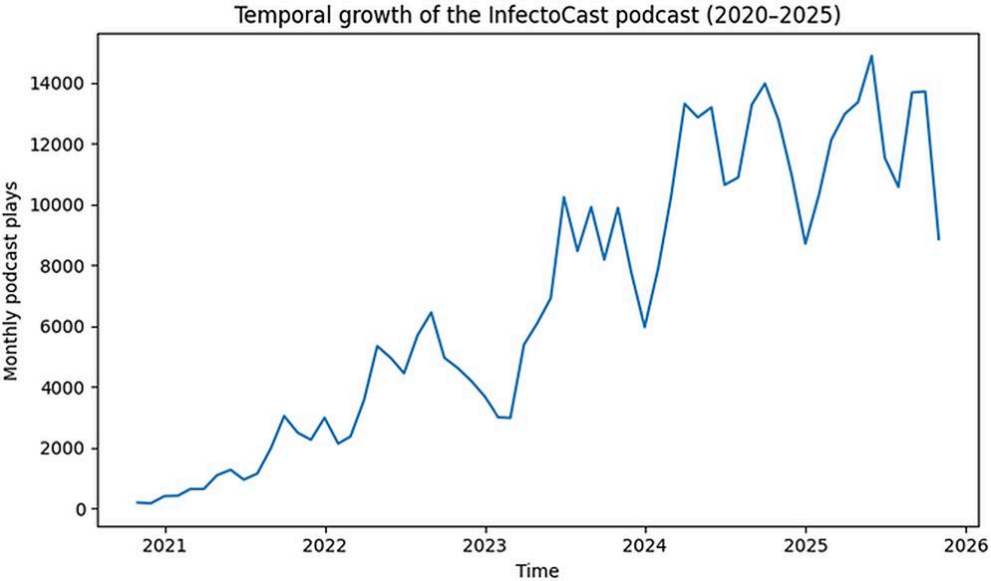
Temporal growth of the InfectoCast podcast (2020–2025). Monthly podcast plays from September 2020 to October 2025. The figure demonstrates sustained growth in listenership over time, with progressive increases in monthly plays as the podcast expanded its content production and integration within the broader digital ecosystem.

Between September 2020 and October 2025, the InfectoCast podcast released a total of 168 episodes. Over this period, the podcast accumulated 481 000 total plays and 53 962 hours of listening time. Monthly podcast plays demonstrated sustained growth, corresponding to a 10 502% increase compared with baseline levels at the beginning of the observation period (Figure 2).

The audience was geographically distributed across 97 countries, with Brazil accounting for 96.9% of total plays. Other countries with notable listenership included the United States, Portugal, and Argentina, each contributing less than 1% of total plays (Table 1). Most listeners accessed the podcast via mobile devices (95.5%), and Spotify represented the primary listening platform, accounting for 83.5% of all plays.

**Table 1.** Geographic Distribution

Rank	Country	Percentage of Plays (%)
1	Brazil	96.91
2	United States	0.65
3	Portugal	0.54
4	Argentina	0.42
5	Paraguay	0.24
6	Colombia	0.17
7	Bolivia	0.17
8	Germany	0.12
9	Costa Rica	0.08
10	Ecuador	0.07

Episodes addressing highly prevalent infectious syndromes in Brazilian clinical practice consistently ranked among the most accessed. Table 2 presents the 5 most listened-to episodes, including community-acquired pneumonia, syphilis, dengue, leptospirosis, and skin and soft tissue infections, with individual episode play counts ranging from approximately 5200 to over 8100.

**Table 2.** Top 5 Podcast Episodes

Rank	Episode Title (Translated)	Plays
1	#17—Community-Acquired Pneumonia	8151
2	#8—Syphilis	8023
3	#37—Dengue	6090
4	#30—Leptospirosis	5946
5	#20—Cellulitis and Erysipelas	5208

### Social Media Platforms and Audience Reach

The social media component of the InfectoCast ecosystem demonstrated substantial reach and engagement across multiple platforms. During the most recent consolidated analysis period (September 2025 to February 2026), the combined reach across platforms exceeded 2.7 million users, with marked increases in engagement metrics compared with the preceding period.

Instagram emerged as the central hub for visual science communication. During the same period, the account achieved more than 2.7 million impressions, over 154 000 interactions, and sustained growth in followers. Engagement indicators included likes, comments, saves, and shares, reflecting active audience interaction with educational content.

Other platforms contributed complementary reach. The YouTube channel accumulated 96 478 views, 2720 subscribers, and more than 2300 hours of watch time, with short-form educational videos accounting for a substantial proportion of recent growth. Additional dissemination occurred through X (formerly Twitter), and LinkedIn, which primarily supported amplification and cross-posting of content.

A consolidated overview of platforms and corresponding audience metrics is presented in Table 3.

**Table 3.** Platforms and Metrics

Platform	Primary Content Format	Key Metrics	Observation Period
Podcast (InfectoCast)	Long-form audio episodes (expert interviews, case discussions, evidence-based reviews)	481 000 total plays; 53 962 listening hours; 168 episodes; audience in 97 countries; 96.9% of plays from Brazil	Sep 2020—Oct 2025
Instagram (@infectocast)	Infographics, educational carousels, Reels, interactive Stories	>2.7 million impressions; >154 000 interactions; 110 000 followers; interaction rates 3%–8% depending on format	Sep 2025—Feb 2026
YouTube (InfectoCast)	Video episodes, short educational videos (Shorts), recorded lectures	96 478 total views; 2720 subscribers; >2300 h of watch time; average view duration ∼5 min	Sep 2025—Feb 2026
Newsletter (“Dose de Ataque”)	Weekly curated written content (clinical updates, literature highlights)	∼9000 subscribers per edition; open rates ranging from 26.0% to 35.2%	Jan 2025—Feb 2026
Blog/website	Long-form written content, show notes, clinical guides	Qualitative repository of searchable educational resources; quantitative access metrics not consistently available	Continuous
X (formerly Twitter)	Short-form updates, scientific commentary, amplification of content	Platform-level impressions and engagement used for content dissemination; historical metrics limited	Continuous
LinkedIn	Professional updates, long-form posts, academic communication	Used primarily for professional outreach and dissemination; quantitative metrics not consistently available	Continuous

### Visual Content Strategy and Engagement Patterns on Instagram

Instagram content was intentionally structured around multiple educational formats, including infographics, carousels, short-form videos (Reels), and interactive Stories. Carousels and infographics accounted for the highest absolute reach, frequently exceeding 50 000 impressions per post, while maintaining interaction rates between 3% and 8%. Reels demonstrated rapid growth during the observation period, with marked increases in reach, shares, and saves, indicating strong engagement with concise, visually driven educational messages.

Interactive Stories contributed additional engagement through polls, questions, and short quizzes, with retention rates frequently exceeding 60%. Table 4 summarizes the educational objectives and average performance metrics associated with each visual content format.

**Table 4.** Visual Content Strategy on Instagram

Content Format	Educational Objective
Infographics	Summaries of guidelines, pathogen biology, mechanisms of drug action.
Carousels	Step-by-step cases, diagnostic reasoning, therapeutic algorithms.
Reels (short videos)	Quick clinical pearls, myth-busting, brief evidence-based tips.
Interactive Stories	Polls, quizzes, Q&A sessions, micro-feedback from the audience.

### Newsletter Performance

The InfectoCast newsletter (“Dose de Ataque”) demonstrated consistent engagement across recent editions. Among the most recent issues, newsletters were delivered to approximately 9000 subscribers per edition, with open rates ranging from 26.0% to 35.2%. The highest open rates were observed in editions addressing clinically focused topics, such as corticosteroid use in pneumonia, diagnostic challenges in ID, and emerging viral infections.

These open rates are comparable to or exceed commonly reported benchmarks for medical and scientific newsletters, supporting the newsletter's role as a complementary dissemination channel within the broader ecosystem.

### Cross-Platform Content Reinforcement

Temporal associations were observed between podcast episode releases and synchronized dissemination through social media and newsletter channels. Episodes accompanied by Instagram carousels or short videos frequently coincided with short-term peaks in daily podcast plays. While causal relationships cannot be established within the scope of this descriptive analysis, these patterns suggest that coordinated, multi-platform dissemination were temporally associated with increased audience attention.

### Overall Ecosystem Reach

When combining podcast listenership, social media impressions, video views, blog access, and newsletter delivery, the InfectoCast ecosystem achieved an estimated reach of approximately 3 million users per month during peak periods. This integrated, multi-platform structure provided multiple entry points for engagement with ID educational content across audio, visual, and written formats.

## DISCUSSION

This study provides a descriptive characterization of a large, Portuguese-language digital ecosystem dedicated to ID education and science communication. To our knowledge, this is one of the first reports to systematically describe the structure, reach, and multi-platform integration of an ID-focused educational initiative developed for a non-Anglophone context. The findings document sustained audience growth across multiple digital channels and illustrate how different media formats can be combined to expand access to educational content.

### The Role of Social Media in Contemporary Medical Education

Our results are consistent with existing literature highlighting the growing role of social media as a complementary educational resource in medicine [4–6]. With a substantial following on Instagram and high monthly reach across platforms, InfectoCast exemplifies how visual microlearning formats—such as infographics, carousels, and short-form videos—can be used to disseminate complex scientific information in accessible ways.

Instagram, in particular, enables rapid distribution of visually structured content, allowing users to engage with topics such as antimicrobial mechanisms, diagnostic reasoning, and emerging infectious threats in a concise format. These characteristics align with prior evidence supporting multimodal learning and the educational value of combining visual and textual elements [7].

Although direct measures of learning outcomes were not assessed, engagement metrics such as interactions, saves, shares, and comments indicate active user interaction with the content. These features suggest that social media platforms may facilitate informal knowledge exchange and ongoing engagement with educational material, consistent with principles described in social and collaborative learning frameworks [5, 9].

### Podcasting Within a Broader Digital Ecosystem

Podcast-based education has gained increasing relevance within ID training and continuing education. The evaluation of the Febrile podcast by Dong and Stead [10] demonstrated that freely accessible audio resources can support asynchronous learning and are well received by both trainees and practicing clinicians. Similar to these initiatives, InfectoCast demonstrates that podcasting can achieve substantial reach when embedded within a broader digital ecosystem.

In this context, the podcast functions as the primary long-form educational component, while social media, written resources, and video-based content provide complementary formats that reinforce and extend the core material. This integrated structure supports repeated exposure to key concepts across different modalities, accommodating diverse learning preferences without replacing formal educational curricula.

### An Integrated and Scalable Educational Model

A defining characteristic of InfectoCast is its ecosystem-based architecture. Rather than operating as isolated platforms, its components are designed to function in a complementary manner: podcast episodes introduce topics in depth; visual content summarizes and reinforces key points; written materials provide expanded discussion; and support tools offer reference information. This multimodal configuration reflects established principles of multimedia learning and distributed repetition [7].

Importantly, this study does not evaluate the impact of the ecosystem on clinical behavior or patient outcomes. However, the prominence of content related to common infectious syndromes and antimicrobial use among the most accessed materials suggests alignment between audience interests and prevalent clinical challenges in the Brazilian healthcare setting. These observations support the relevance of locally contextualized educational initiatives in regions where access to specialized ID education may be uneven.

### Positioning InfectoCast Within the Infectious Diseases Landscape

Brazil's epidemiological profile—characterized by endemic arboviral diseases, fungal infections, heterogeneous antimicrobial resistance patterns, and a geographically diverse healthcare system—underscores the need for educational resources tailored to local contexts. Most digital ID education initiatives operate primarily in English and may not address region-specific epidemiology or health system realities.

By delivering content in Portuguese and framing discussions around local clinical and public health priorities, InfectoCast addresses a recognized gap in access to continuing ID education. The scale and sustained growth documented in this analysis indicate demand for such resources, particularly in non-Anglophone settings.

### Limitations

This study has several limitations. First, it relies exclusively on aggregated, platform-generated analytics, which limits the ability to assess educational effectiveness, knowledge acquisition, behavior change, or impact on clinical practice. Statements regarding clinical relevance should therefore be interpreted as descriptive rather than evaluative.

Second, while podcast and Instagram analytics were available in detail, metrics from other platforms—such as X, LinkedIn, and YouTube—were less comprehensive, restricting cross-platform comparisons. Third, the analysis represents a snapshot of a continuously evolving ecosystem; engagement patterns and platform roles may change over time as new formats and tools are introduced.

Future research should incorporate qualitative methods, learner surveys, or mixed-methods designs to more formally evaluate educational outcomes and explore alignment with established evaluation frameworks, such as the Kirkpatrick model.

### Conclusions

This descriptive case report documents the structure, reach, and growth of InfectoCast, a Portuguese-language digital ecosystem for ID education and science communication. The initiative demonstrates how integrating podcasting with social media, video, and written content can expand access to educational materials in a non-Anglophone context.

Rather than evaluating educational impact, this study provides a transparent account of ecosystem design and audience engagement, offering a reproducible model for other medical specialties and regions facing similar linguistic and contextual barriers. Future evaluative work is needed to assess learning outcomes and potential effects on clinical practice, but the present findings highlight the feasibility and scalability of ecosystem-based digital education in ID.

The podcast is available on Spotify and other streaming platforms, and more information can be found at https://instagram.com/infectocast.english and https://www.instagram.com/infectocast.espanol.
